# Supplementary material for: Chromosomal Instability Is Associated with cGAS–STING Activation in EGFR-TKI Refractory Non-Small-Cell Lung Cancer
Source: Cells. 2025 Mar 17;14(6):447. doi: 10.3390/cells14060447 (PMC11941500; doi:10.3390/cells14060447)
Supplement: Supplementary file 1 [file cells-14-00447-s001.zip › Supple methods.yonesaka_2025.2.8.pdf]

Supplementary Methods

DNA panel sequencing

As previously described [29], a DNA panel sequencing was conducted. The library preparation was conducted using an Illumina TruSight Oncology 500 Kit (TSO500, Illumina Inc., San Diego, CA, USA). A DNA input of 110 ng was utilized for library preparation; however, the maximum DNA input was employed when the amount of DNA was insufficient. qPCR was conducted using the QuantStudio 12 K Flex Real-Time PCR System (Thermo Fisher Scientific Inc.). To fragment the DNA strands into fragments of 90–250 base pairs, the DNA of each sample was subjected to shearing using the Covaris E220 ultrasonicator (Covaris, Inc., Woburn, MA, USA) [29]. The normalized libraries were subsequently sequenced using the NextSeq 500 system (Illumina, Inc.). The sequencing reads were aligned to the human reference genome (hg19), after which mutation call analyses were performed using TSO500 analysis software (v1.3.1) and annotated using Variant Effect Predictor (VEP, release_98.3). These analyses included single nucleotide variants (SNVs) and insertion/deletion (InDel) mutations. Copy number variation (CNV) analyses were conducted using TSO500 analysis software versions 1.3.1 and 2.1, respectively. Samples with a low library concentration (<3 ng/μL) were excluded, as were SNVs with a low mutant allele count (<5). Additionally, germline mutations known from the Human Genetic Variation Database were excluded.

RNA panel sequencing

As previously described [29], an RNA panel sequencing was conducted. The library was prepared using TSO500, in accordance with the instructions provided by the manufacturer. A total of 170 ng of RNA was utilized for library preparation; however, in instances where the

RNA quantity was inadequate, the maximum RNA input was employed. The normalized libraries were sequenced to a length of 100 base pairs (2 × 100 bp) from both ends using the NextSeq, NextSeq500, or NextSeq550 sequencing system (Illumina, Inc.). The sequencing reads were aligned to a human sequence (hg19). Fusion and splice variant calls were made using the TST170 analysis software, version 2.0.0. Samples with insufficient library concentration (less than 3 ng/μL) were excluded from the dataset.

Transcriptional profiling

RNA was extracted from formalin-fixed paraffin-embedded (FFPE) tumors that exhibited a cellular composition comprising more than 50% cancer cells. The cDNA library for RNA-seq was prepared using the directional sequencing method, which included ribosomal RNA depletion and the incorporation of unique dual indexes. The requisite kits and reagents for this process were as follows: The following kits and reagents were utilized: QIAseq FastSelect (Qiagen Inc., Venlo, Netherlands), NEBNext Ultra II Directional RNA Library Prep Kit for Illumina (New England Biolabs, Inc., Ipswich, MA, USA), and NEBNext Multiplex Oligos for Illumina (New England Biolabs, Inc.). An RNA input of 20 ng was utilized for library preparation; however, in the event that the quantity of RNA was insufficient, the maximum RNA input was employed. The quantity of the prepared library was evaluated using the Agilent 4200 TapeStation. The prepared libraries were pooled in a single tube and sequenced on a single flow cell at 1.5 nM using a NovaSeq 6000 (Illumina, Inc.), with 75 bp from both ends (2 × 75 bp). The sequencing reads were aligned with the STAR software (version 2.5.3a) to the human genome reference (GRCh38). The transcripts per million and expected count for each gene were estimated using the RNA-seq and expectation maximization (RSEM) software (1.3.0). Samples comprising non-cancerous cells or exhibiting a low sum of expected counts were excluded and subjected to

normalization through the application of the trimmed mean of M-value normalization. For the purposes of data analysis, samples exhibiting less than 50% of the total reads within a given species or a total of less than 50 million reads were excluded.

siRNA transfection

siRNA against cGAS (MB21D1, No. 115004) was purchased from Horizon Discovery (USA). It consisted of 21-nucleotide sense and antisense strands. Non-targeting siRNA (siMock) was used as the nonspecific control. H1975 cells were seeded at 50% confluence in six-well plates, incubated for 24 h in RPMI-1640 medium supplemented with 0.5% FBS, and transfected with siRNAs using Lipofectamine RNAiMax (Invitrogen, Carlsbad, CA, USA). After 48 h, cells were harvested for western blotting.
