# Supplementary material for: Chromosomal Instability Is Associated with cGAS–STING Activation in EGFR-TKI Refractory Non-Small-Cell Lung Cancer
Source: Cells. 2025 Mar 17;14(6):447. doi: 10.3390/cells14060447 (PMC11941500; doi:10.3390/cells14060447)
Supplement: Supplementary file 1 [file cells-14-00447-s001.zip › Supplement Figure 1_2025.3.1.pptx]

## Slide 1
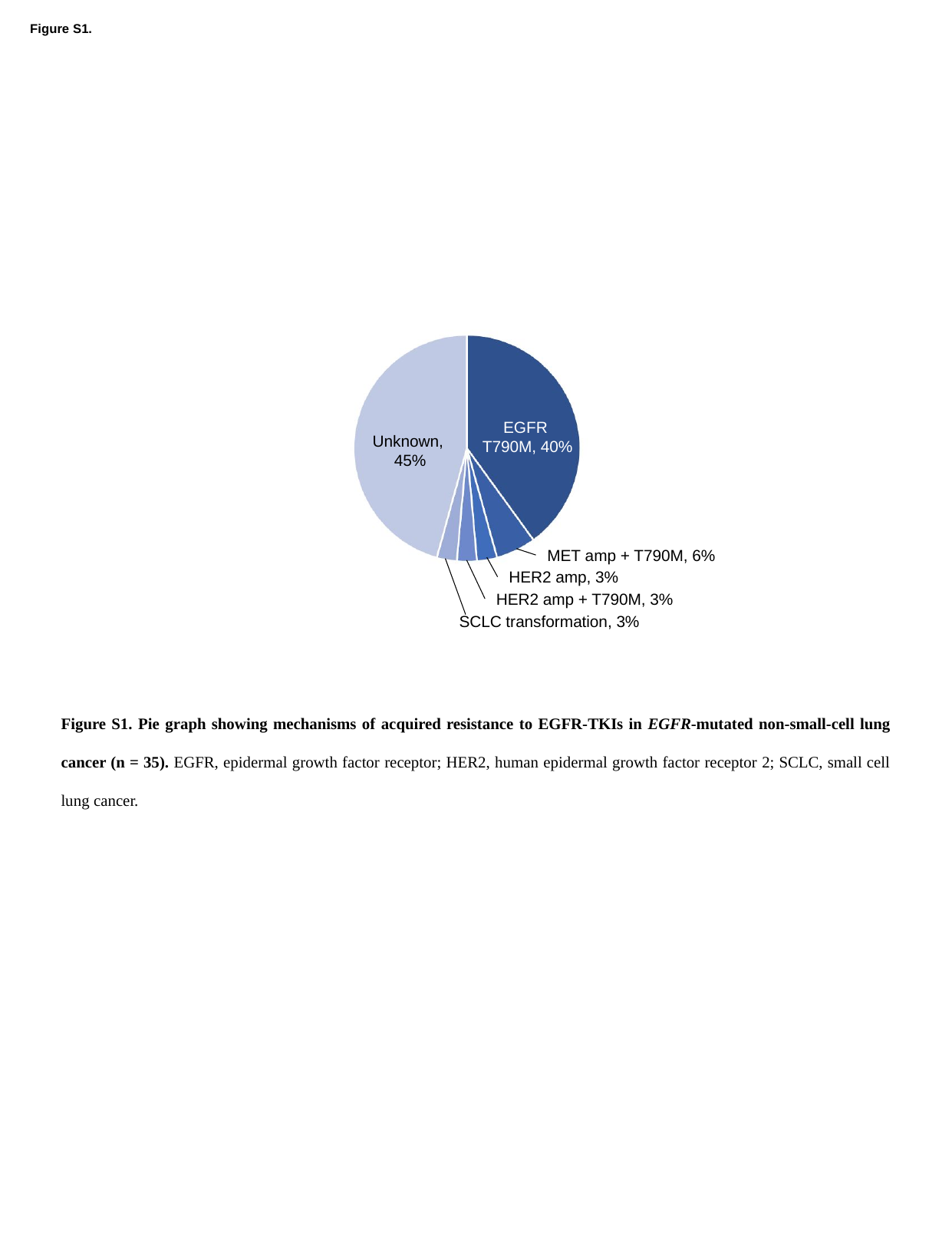

Figure S1.
EGFR
T790M, 40%
Unknown,
45%
MET amp + T790M, 6%
HER2 amp, 3%
HER2 amp + T790M, 3%
SCLC transformation, 3%
Figure S1. Pie graph showing mechanisms of acquired resistance to EGFR-TKIs in EGFR-mutated non-small-cell lung cancer (n = 35). EGFR, epidermal growth factor receptor; HER2, human epidermal growth factor receptor 2; SCLC, small cell lung cancer.
