# Supplementary material for: Chromosomal Instability Is Associated with cGAS–STING Activation in EGFR-TKI Refractory Non-Small-Cell Lung Cancer
Source: Cells. 2025 Mar 17;14(6):447. doi: 10.3390/cells14060447 (PMC11941500; doi:10.3390/cells14060447)
Supplement: Supplementary file 1 [file cells-14-00447-s001.zip › Supplement Figure 2.pptx]

## Slide 1
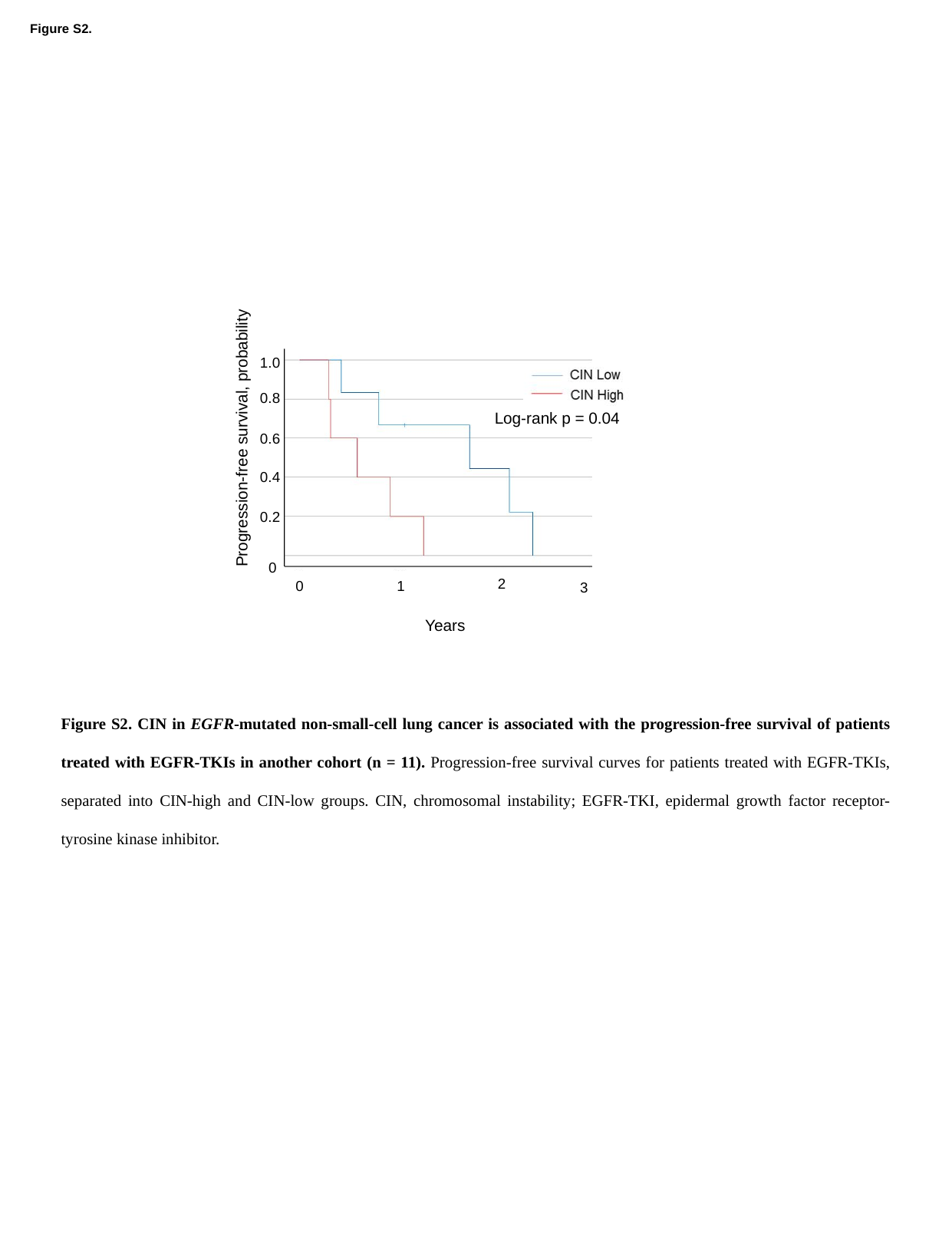

Figure S2.
1.0
0.8
Log-rank p = 0.04
Progression-free survival, probability
0.6
0.4
0.2
0
2
0
1
3
Years
Figure S2. CIN in EGFR-mutated non-small-cell lung cancer is associated with the progression-free survival of patients treated with EGFR-TKIs in another cohort (n = 11). Progression-free survival curves for patients treated with EGFR-TKIs, separated into CIN-high and CIN-low groups. CIN, chromosomal instability; EGFR-TKI, epidermal growth factor receptor-tyrosine kinase inhibitor.
