# Supplementary material for: Chromosomal Instability Is Associated with cGAS–STING Activation in EGFR-TKI Refractory Non-Small-Cell Lung Cancer
Source: Cells. 2025 Mar 17;14(6):447. doi: 10.3390/cells14060447 (PMC11941500; doi:10.3390/cells14060447)
Supplement: Supplementary file 1 [file cells-14-00447-s001.zip › Supplement Figure 4.pptx]

## Slide 1
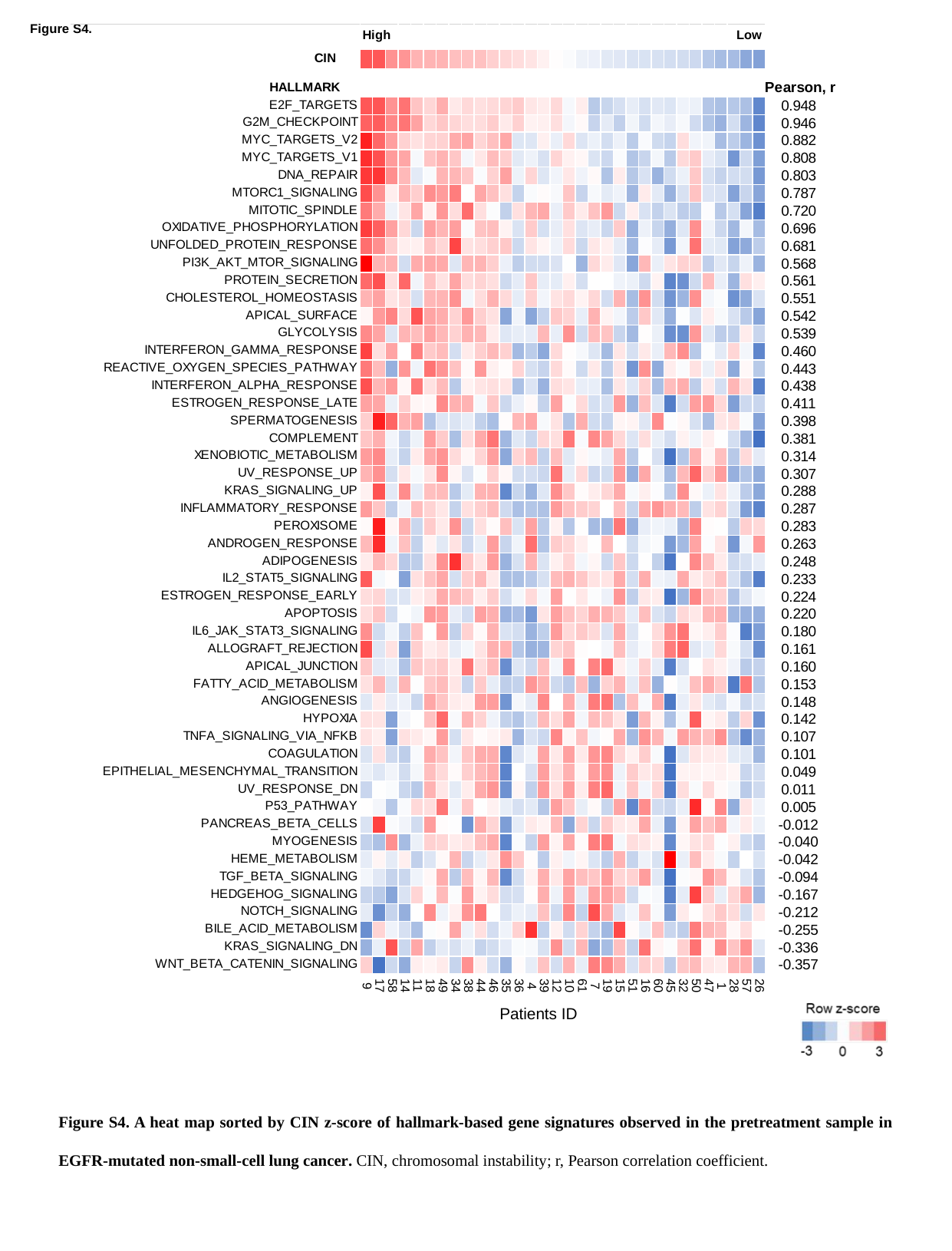

Figure S4.
Patients ID
Figure S4. A heat map sorted by CIN z-score of hallmark-based gene signatures observed in the pretreatment sample in EGFR-mutated non-small-cell lung cancer. CIN, chromosomal instability; r, Pearson correlation coefficient.
