# Supplementary material for: Chromosomal Instability Is Associated with cGAS–STING Activation in EGFR-TKI Refractory Non-Small-Cell Lung Cancer
Source: Cells. 2025 Mar 17;14(6):447. doi: 10.3390/cells14060447 (PMC11941500; doi:10.3390/cells14060447)
Supplement: Supplementary file 1 [file cells-14-00447-s001.zip › Supplement Figure 5.pptx]

## Slide 1
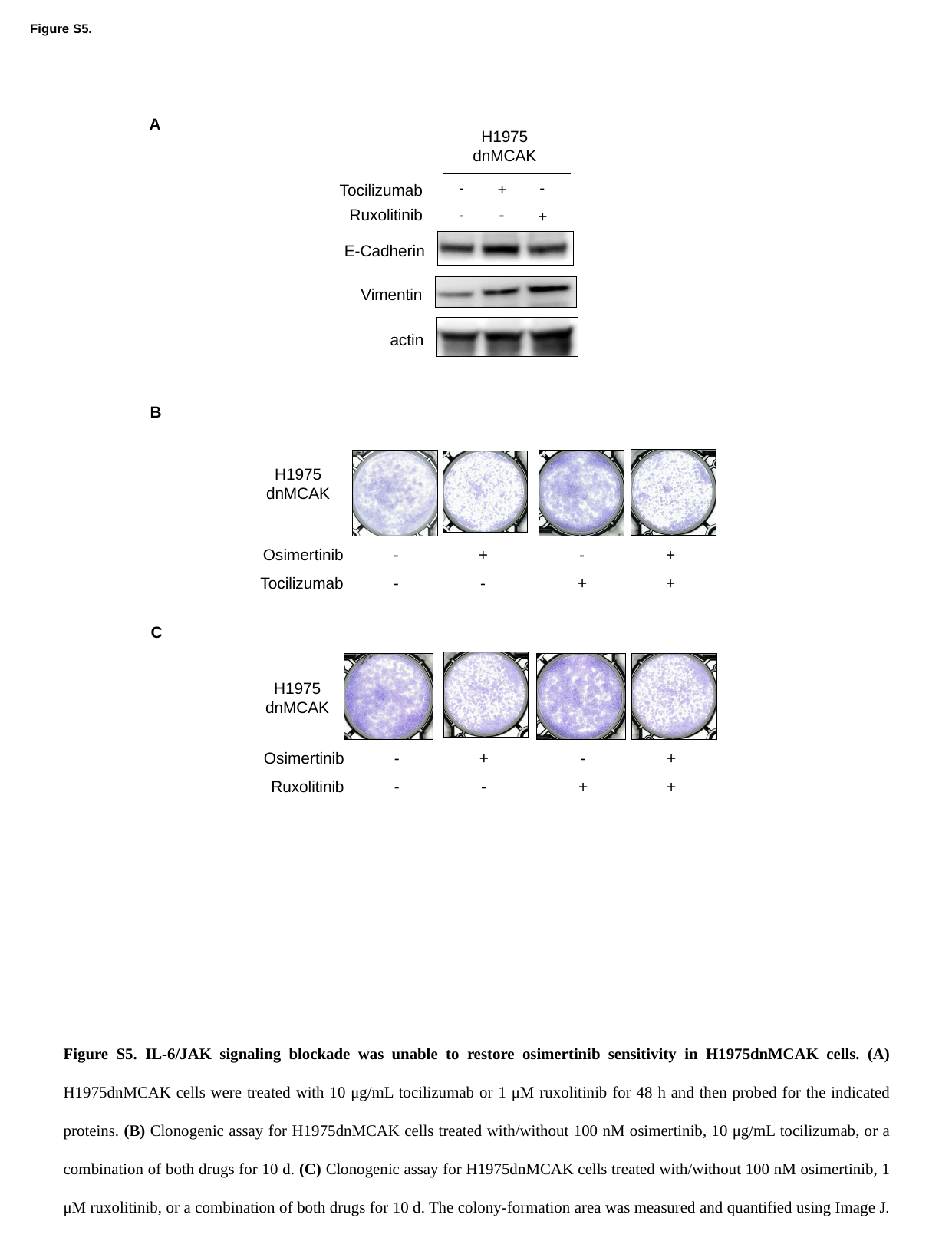

Figure S5.
A
H1975
dnMCAK
-
-
+
Tocilizumab
-
-
Ruxolitinib
+
E-Cadherin
Vimentin
actin
B
H1975
dnMCAK
Osimertinib
-
+
-
+
Tocilizumab
-
-
+
+
C
H1975
dnMCAK
Osimertinib
-
+
-
+
Ruxolitinib
-
-
+
+
Figure S5. IL-6/JAK signaling blockade was unable to restore osimertinib sensitivity in H1975dnMCAK cells. (A) H1975dnMCAK cells were treated with 10 μg/mL tocilizumab or 1 μM ruxolitinib for 48 h and then probed for the indicated proteins. (B) Clonogenic assay for H1975dnMCAK cells treated with/without 100 nM osimertinib, 10 μg/mL tocilizumab, or a combination of both drugs for 10 d. (C) Clonogenic assay for H1975dnMCAK cells treated with/without 100 nM osimertinib, 1 μM ruxolitinib, or a combination of both drugs for 10 d. The colony-formation area was measured and quantified using Image J.
