# Supplementary material for: Chromosomal Instability Is Associated with cGAS–STING Activation in EGFR-TKI Refractory Non-Small-Cell Lung Cancer
Source: Cells. 2025 Mar 17;14(6):447. doi: 10.3390/cells14060447 (PMC11941500; doi:10.3390/cells14060447)
Supplement: Supplementary file 1 [file cells-14-00447-s001.zip › Supplement Figure 6.pptx]

## Slide 1
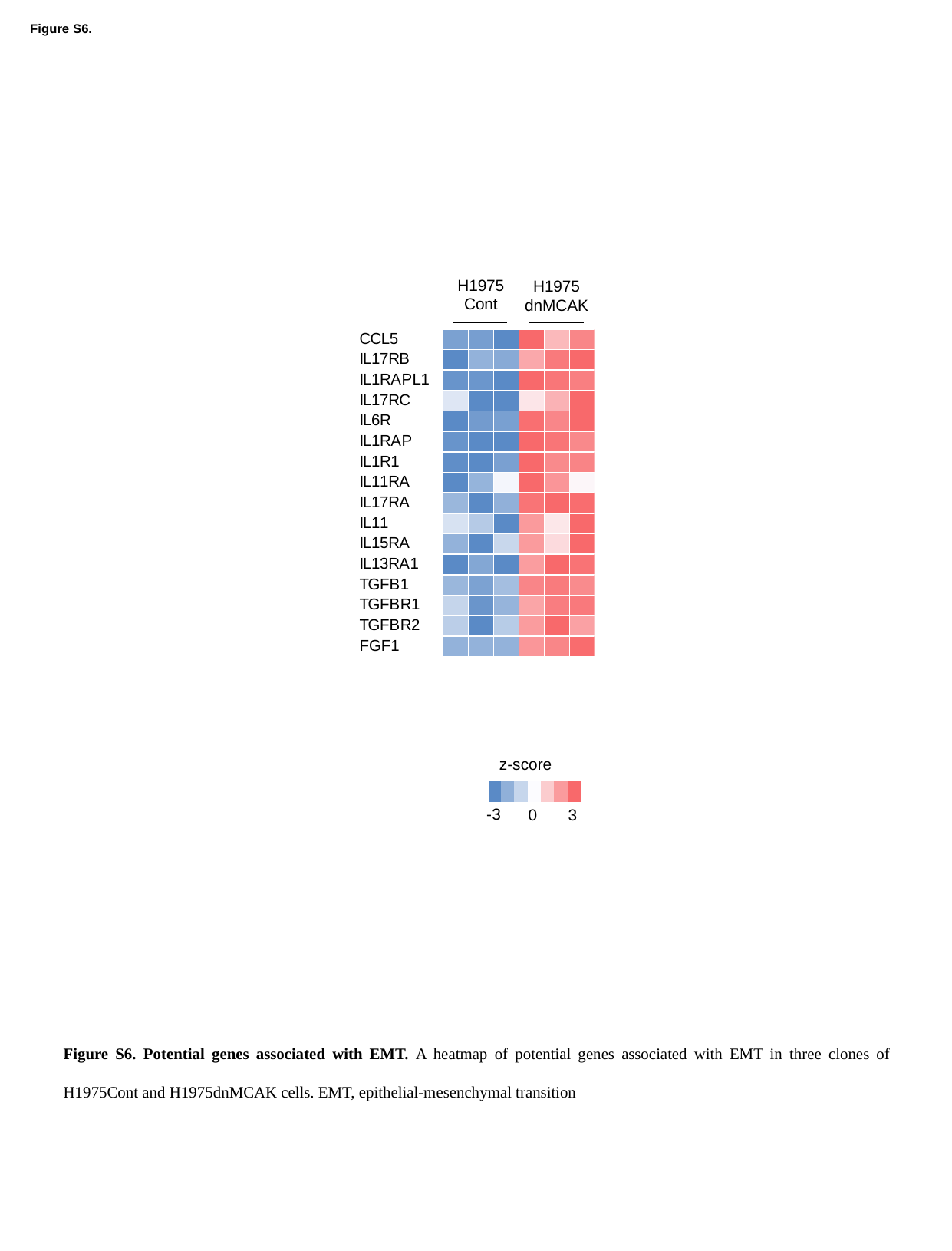

Figure S6.
H1975
Cont
H1975
dnMCAK
z-score
-3
0
3
Figure S6. Potential genes associated with EMT. A heatmap of potential genes associated with EMT in three clones of H1975Cont and H1975dnMCAK cells. EMT, epithelial-mesenchymal transition
