# Supplementary material for: Chromosomal Instability Is Associated with cGAS–STING Activation in EGFR-TKI Refractory Non-Small-Cell Lung Cancer
Source: Cells. 2025 Mar 17;14(6):447. doi: 10.3390/cells14060447 (PMC11941500; doi:10.3390/cells14060447)
Supplement: Supplementary file 1 [file cells-14-00447-s001.zip › Supplement Figure 7_2025.3.1.pptx]

## Slide 1
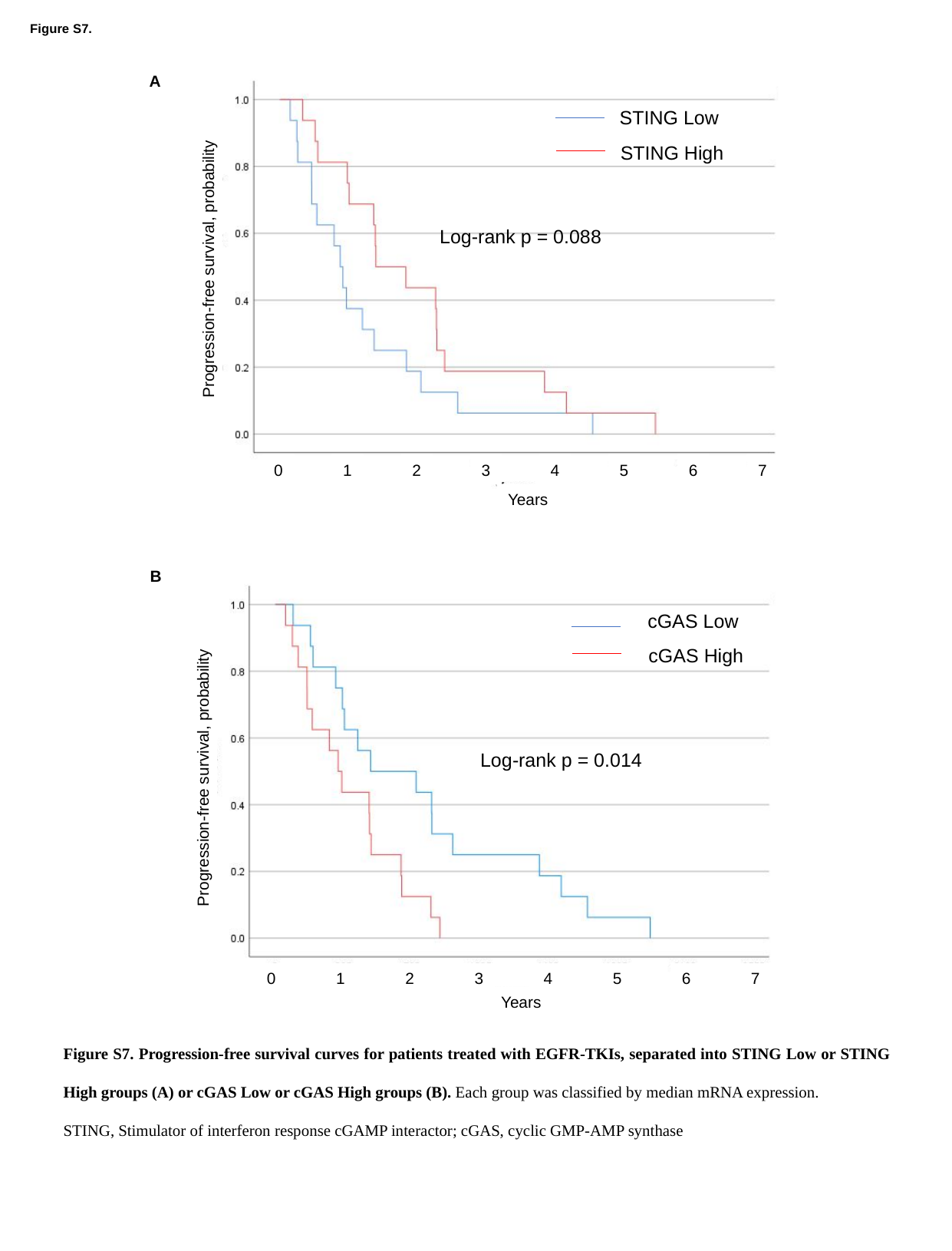

Figure S7.
A
STING Low
STING High
Log-rank p = 0.088
0
1
2
3
4
5
6
7
Years
A
Progression-free survival, probability
cGAS Low
cGAS High
Log-rank p = 0.014
Progression-free survival, probability
0
1
2
3
4
5
6
7
Years
B
Figure S7. Progression-free survival curves for patients treated with EGFR-TKIs, separated into STING Low or STING High groups (A) or cGAS Low or cGAS High groups (B). Each group was classified by median mRNA expression.
STING, Stimulator of interferon response cGAMP interactor; cGAS, cyclic GMP-AMP synthase
