# Supplementary material for: Chromosomal Instability Is Associated with cGAS–STING Activation in EGFR-TKI Refractory Non-Small-Cell Lung Cancer
Source: Cells. 2025 Mar 17;14(6):447. doi: 10.3390/cells14060447 (PMC11941500; doi:10.3390/cells14060447)
Supplement: Supplementary file 1 [file cells-14-00447-s001.zip › Supplement Figure 8_2025.3.5.pptx]

## Slide 1
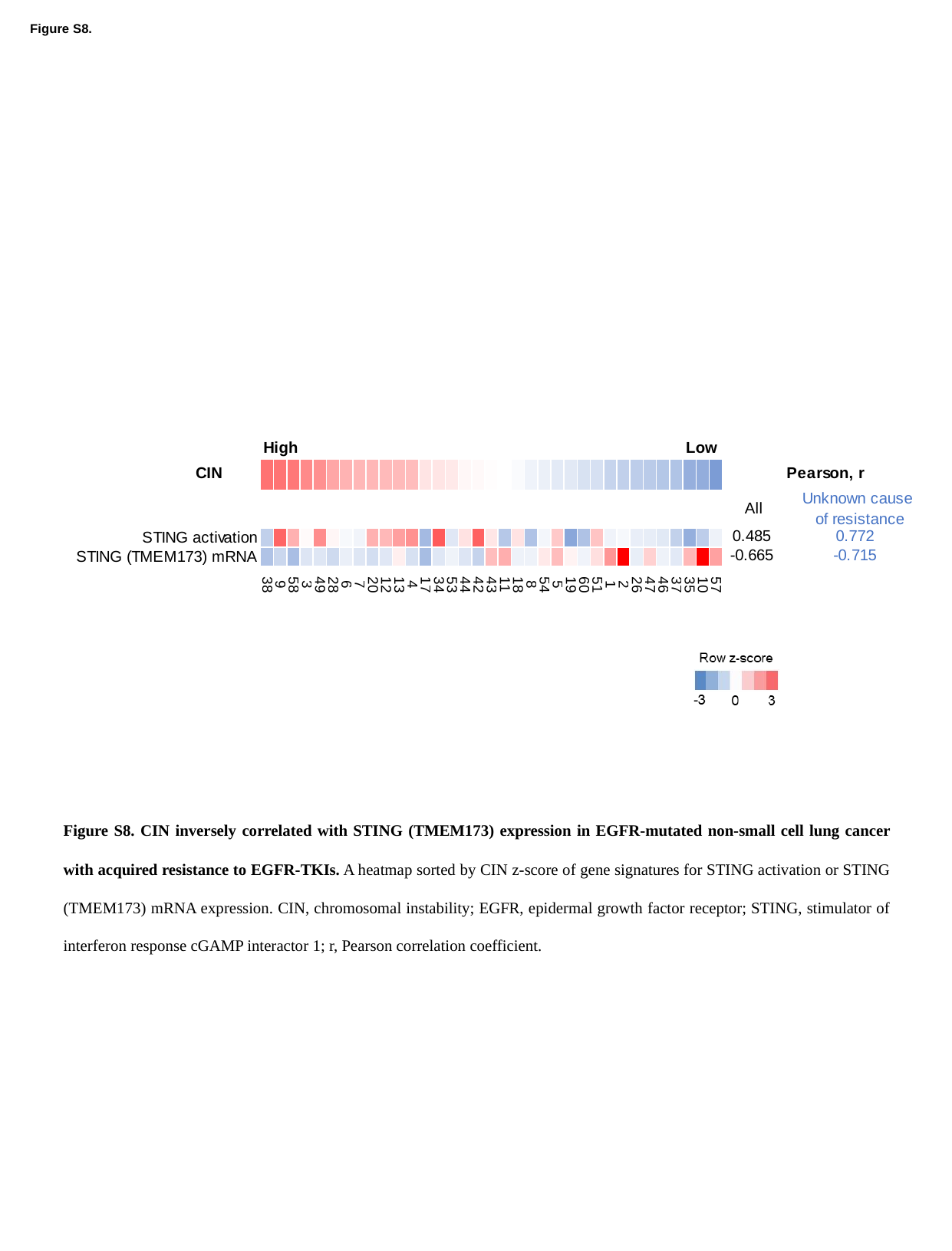

Figure S8.
Figure S8. CIN inversely correlated with STING (TMEM173) expression in EGFR-mutated non-small cell lung cancer with acquired resistance to EGFR-TKIs. A heatmap sorted by CIN z-score of gene signatures for STING activation or STING (TMEM173) mRNA expression. CIN, chromosomal instability; EGFR, epidermal growth factor receptor; STING, stimulator of interferon response cGAMP interactor 1; r, Pearson correlation coefficient.
