# Supplementary material for: Chromosomal Instability Is Associated with cGAS–STING Activation in EGFR-TKI Refractory Non-Small-Cell Lung Cancer
Source: Cells. 2025 Mar 17;14(6):447. doi: 10.3390/cells14060447 (PMC11941500; doi:10.3390/cells14060447)
Supplement: Supplementary file 1 [file cells-14-00447-s001.zip › Supplement Table S1.pptx]

## Slide 1
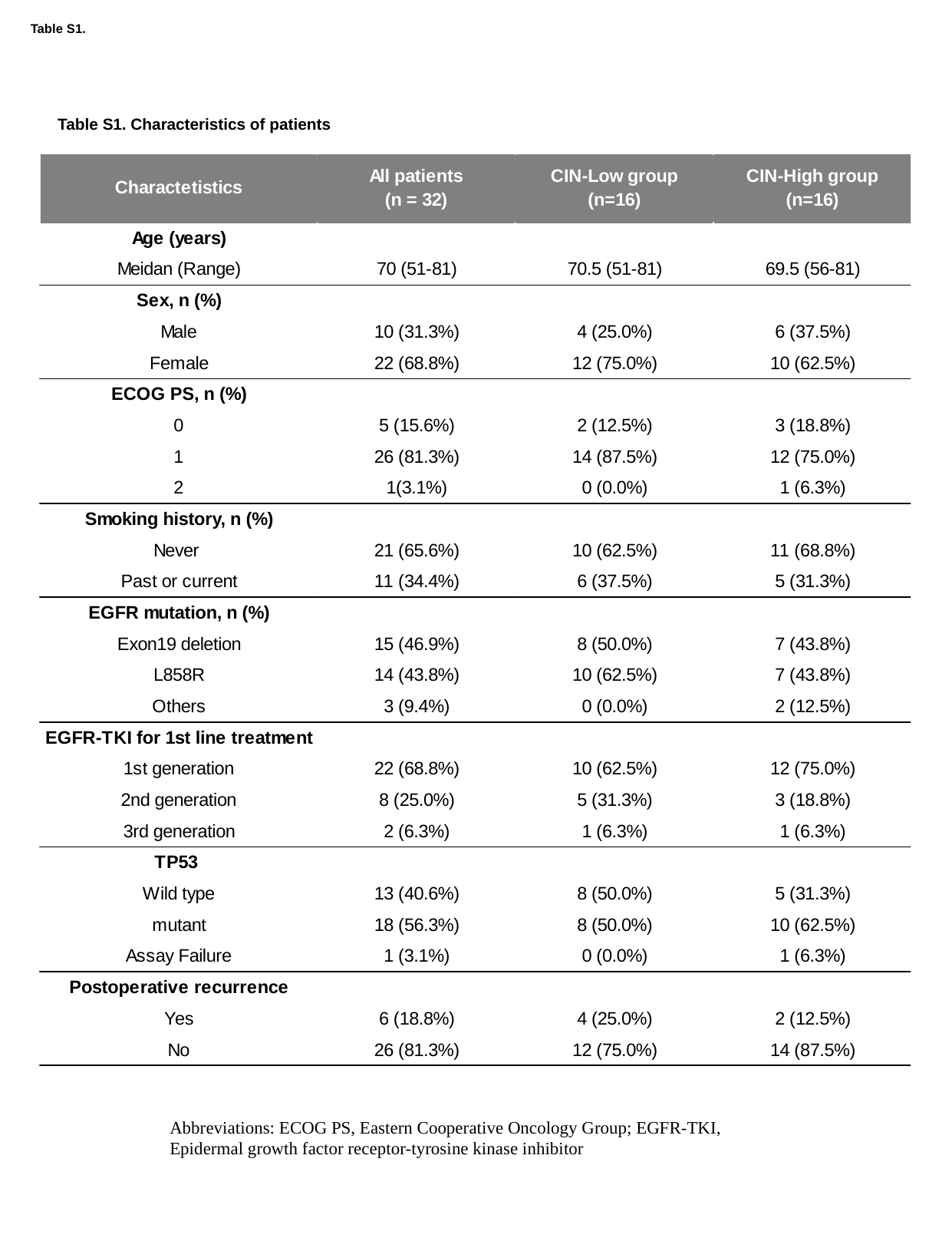

Table S1.
Table S1. Characteristics of patients
Abbreviations: ECOG PS, Eastern Cooperative Oncology Group; EGFR-TKI, Epidermal growth factor receptor-tyrosine kinase inhibitor
