# Supplementary figures and images for: Chromosomal Instability Is Associated with cGAS–STING Activation in EGFR-TKI Refractory Non-Small-Cell Lung Cancer
Source: Cells. 2025 Mar 17;14(6):447. doi: 10.3390/cells14060447 (PMC11941500; doi:10.3390/cells14060447)

## Slide 1
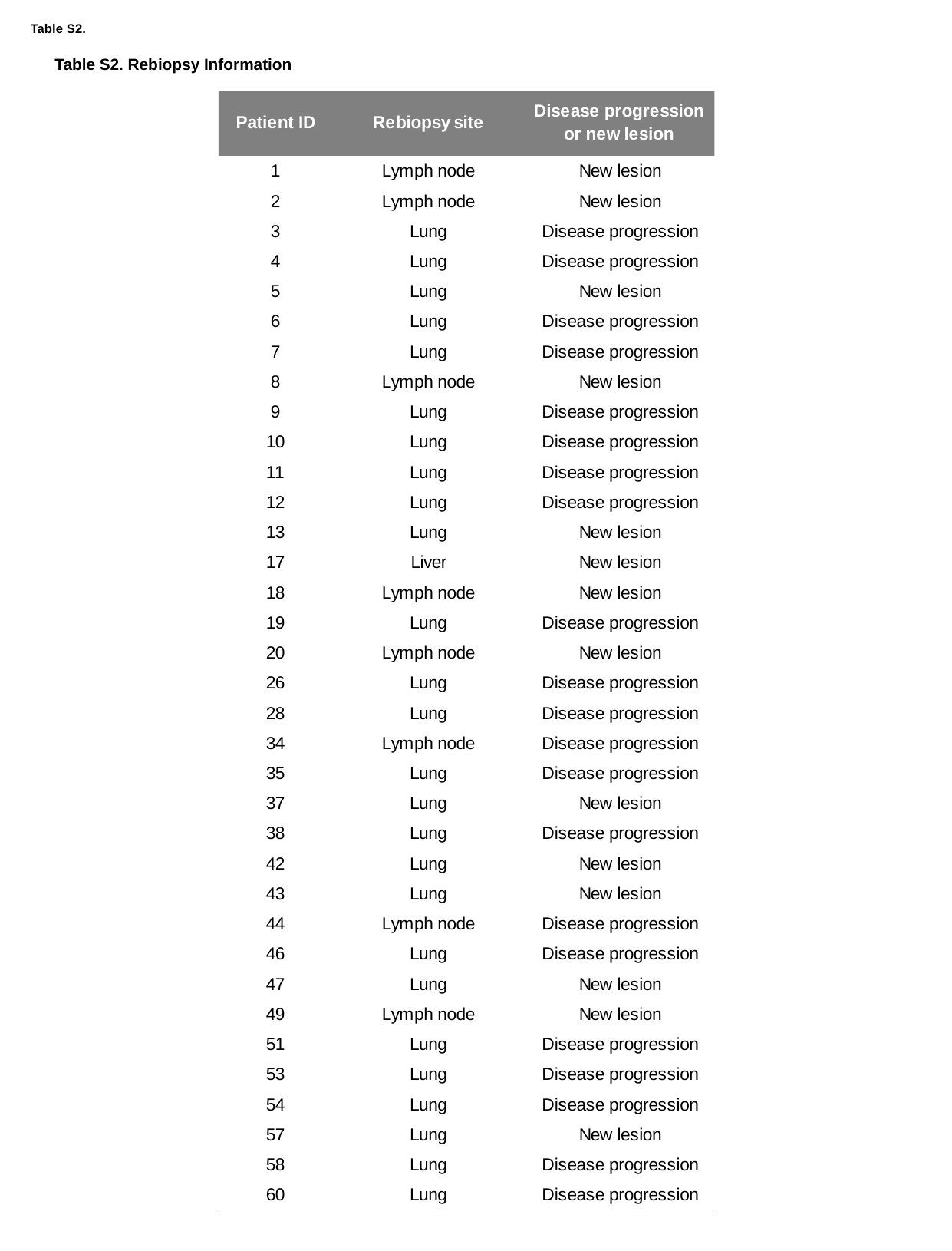

Table S2.
Table S2. Rebiopsy Information

Supplement: Supplementary file 1 [file cells-14-00447-s001.zip › Supplement Table S2.pptx]
